# Predictive Value of ^18^F-FDG PET and CT Morphologic Features for Recurrence in Pathological Stage IA Non-Small Cell Lung Cancer

**DOI:** 10.1097/MD.0000000000000434

**Published:** 2015-01-26

**Authors:** Kai-Hsiung Ko, Hsian-He Hsu, Tsai-Wang Huang, Hong-Wei Gao, Cheng-Yi Cheng, Yi-Chih Hsu, Wei-Chou Chang, Chi-Ming Chu, Jia-Hong Chen, Shih-Chun Lee

**Affiliations:** From the Department of Radiology (K-HK, H-HH, Y-CH, W-CC); Department of Thoracic Surgery (T-WH, S-CL); Department of Pathology (H-WG); Department of Nuclear Medicine (C-YC), Tri-Service General Hospital, National Defense Medical Center; Section of Health Informatics (C-MC), Institute of Public Health, National Defense Medical Center; and Division of Hematology-Oncology (J-HC), Department of Internal Medicine, Tri-Service General Hospital, National Defense Medical Center, Taipei, Taiwan, Republic of China.

## Abstract

Patients with pathological stage IA non-small cell lung cancer (NSCLC) may relapse despite complete surgical resection without lymphovascular invasion. A method of selecting a high-risk group for adjuvant therapy is necessary. The aim of this study was to assess the predictive value of ^18^F-fluorodeoxyglucose (FDG) uptake and the morphologic features of computed tomography (CT) for recurrence in pathological stage IA NSCLC.

One hundred forty-five patients with pathological stage IA NSCLC who underwent pretreatment with FDG positron emission tomography and CT evaluations were retrospectively enrolled. The associations among tumor recurrence and patient characteristics, maximal standard uptake value (SUVmax) of primary tumors, and CT imaging features were investigated using univariate and multivariate analyses. A receiver operating characteristic (ROC) curve analysis was performed to quantify the predictive value of these factors.

Tumor recurrence developed in 21 (14.5%) of the 145 patients, and the 5-year recurrence-free survival rate was 77%. The univariate analysis demonstrated that SUVmax, the grade of histological differentiation, tumor size, and the presence of bronchovascular bundle thickening were significant predictive factors (*P* < 0.05). A higher SUVmax (≥2.5) (*P* = 0.021), a lower ground-glass opacity ratio (≤17%) (*P* = 0.014), and the presence of bronchovascular bundle thickening (*P* = 0.003) were independent predictive factors of tumor recurrence in the multivariate analysis. The use of this predictive model yielded a greater area under the ROC curve (0.877), which suggests good discrimination.

The combined evaluation of FDG uptake and CT morphologic features may be helpful in the prediction of recurrence in patients with pathological stage IA NSCLC and in the stratification of a high-risk group for postoperative adjuvant therapy or prospective clinical trials.

## INTRODUCTION

Currently, tumor stage remains the most important factor correlated with survival in non-small cell lung cancer (NSCLC), and the 5-year survival rates range from 83% in pathological stage IA to 23% in stage IIIA tumors.^1,2^ Recently, with the widespread use and advanced technology of computed tomography (CT) for screening and diagnosis, more pathological stage IA tumors have been diagnosed. However, the rate of recurrence remains unsatisfactory and ranges between 15% and 30%, even after complete surgical resection.^2–4^ Many recent studies have recognized that lymphovascular invasion (LVI) is an important and poor prognostic factor for pathological stage I tumors, and adjuvant chemotherapy might be helpful to improve survival in this high-risk group.^5–9^ However, in clinical practice, many cases without LVI still encounter recurrence despite complete surgical resection. This observation encouraged us to investigate whether other risk factors in addition to LVI were correlated with recurrence in pathological stage IA tumors, which will help identify high-risk patients who may benefit from adjuvant chemotherapy and will help in the prediction of outcomes.

^18^F-fluorodeoxyglucose (FDG) positron emission tomography (PET) has been widely used as a noninvasive diagnostic modality for diagnosing, staging, detecting recurrence, and monitoring the therapeutic response in patients with NSCLC.^10^ Furthermore, the degree of FDG uptake of tumor lesions, expressed as the maximal standardized uptake value (SUVmax), is proportional to the glucose metabolic rate of viable tumor cells and is a significant prognostic factor in NSCLC.^11–14^ Thin-slice CT imaging characteristics are also important in the evaluation of malignant potential or pathological aggressiveness of NSCLC. A well-known indicator of invasiveness and a prognostic factor on CT images is the ground-glass opacity (GGO) ratio of early lung cancer.^15,16^ However, it remains unclear whether a relationship exists among FDG uptake, CT imaging features, and recurrence in pathological stage IA NSCLC. Thus, we retrospectively analyzed the clinical and radiological features of pathological stage IA NSCLC patients and developed a prediction model to facilitate the risk assessment of recurrence.

## MATERIALS AND METHODS

The study was approved by the Institutional Review Board for Human Investigation, which waived the requirement of informed consent for this retrospective study. Between January 2005 and December 2011, 472 consecutive patients with NSCLC underwent pulmonary surgery at our institute. The inclusion criteria of the study were as follows: pathological stage IA disease, preoperative CT and FDG PET examinations, and no preoperative or postoperative adjuvant chemotherapy or radiotherapy. Patients with multiple lung cancers or a history of malignancy were excluded. One hundred forty-five patients (48 men and 97 women; mean age, 61.9 years) who met inclusion criteria were enrolled in this study. Clinical parameters, including age, sex, smoking history, pretreatment serum carcinoembryonic antigen (CEA) level (normal range, 0–5 ng/mL), surgical procedure type, pathological findings, and tumor stage, were recorded. Tumor–node metastasis (TNM) staging was performed in all patients based on the 7th edition of the American Joint Committee on Cancer (AJCC) staging manual.^17^ All pathological evaluation, including histological subtype, histological grade, and LVI, was performed by a pathologist with 15 years of experience who was blind to the recurrence status of the patients.

After surgical resection, all patients were followed and assessed by chest CT imaging every 3 to 6 months in the first 2 years and annually thereafter. Recurrence was confirmed by either radiological or pathological exams in all cases. When ambiguous cases occurred, the diagnosis of recurrence was made by the consensus of a multidisciplinary discussion. The recurrence-free survival (RFS) period was calculated in months from the date of surgery until recurrence or the last follow-up.

### PET Acquisition

PET/CT was performed using a Biograph PET/CT scanner (Siemens Inc, Germany). All patients fasted except for water at least 6 hours prior to the examination. The serum glucose level was measured prior to the administration of the radiotracer to ascertain that blood sugar was less than 150 mg/dL. Sixty minutes after the intravenous injection of 10 mCi ^18^F-FDG, imaging was performed using a spiral CT scan from the head to the upper thigh with a 5 mm thickness per slice. Subsequently, PET data were acquired in a supine position using the conventional 3-dimensional mode (field of view, 50 cm in the transaxial plane and 15.5 cm in the axial plane) at 3 minutes per bed position. The CT images were reconstructed using conventional filtered back projection at 2.4 mm axial intervals to match the slice separation of the PET data. The CT attenuation-corrected PET images were reconstructed using the method of ordered subset expectation maximization (2 iterations and 8 subsets). Coregistered images were displayed using SYNGO software (Siemens Inc).

### PET Data Analysis

A nuclear medicine physician with 26 years of experience who was blinded to the recurrent status of the patients interpreted the FDG–PET images. The SUVmax for each patient was measured by placing regions of interest (ROIs) on the axial image of the tumor. The SUVmax was calculated as the maximum FDG uptake within the ROI divided by the injected dose over the patient's body weight (SUVmax = maximum pixel activity/[injected dose/body weight]).

### CT Technique

Chest CT scans (64-detector row scanner [Brilliance; Philips Medical Systems, Cleveland, OH]) from the lung apices through the adrenal glands were available for all patients and had been conducted within 1 month prior to PET scan. The routine scanning parameters were as follows: a slice thickness of 5 mm, a reconstruction interval of 5 mm, a rotation speed of 0.75 seconds, a pitch of 1.05 to 1.25 and 120 kVp, and an effective tube current × time product that ranged between 150 and 200 mA. High-resolution images of the tumor were acquired with a thin slice thickness of 1 mm and reconstructed with a high-spatial-frequency algorithm.

### Analysis of CT Image Features

The CT findings were analyzed in the lung window setting (window level, −700 Hounsfield units [HU]; window width, 1000 HU) using a picture archiving and communication system (PACS) workstation (EBM Technologies Incorporated, Taiwan). All CT images were evaluated in consensus by 2 chest radiologists (H.H.H. and Y.C.H., with 23 and 11 years of experience, respectively) who were aware that all patients had proven lung cancers, but were blinded to the final pathological result and recurrence status. The imaging features that were analyzed for each lesion included the tumor size, tumor location, border (lobulated or nonlobulated), margin (spiculated or nonspiculated), presence of air bronchogram, presence of bronchovascular bundle thickening, presence of pleural retraction, and GGO ratio. GGO was defined as hazy opacity without obscuring the underlying vascular or bronchial structures. Two reviewers also performed quantitative measurements (1 measurement by each reviewer separately) to determine the tumor size and the GGO ratio. The maximal diameter of the entire tumor and internal solid portion on axial images was recorded. The GGO ratio was calculated using the following formula: (1 − [maximal dimension of solid portion/maximal dimension of tumor] × 100%). We used the mean of the 2 measurements (1 measurement by each reviewer separately) as the final result. If the interobserver difference was larger than 5%, a third measurement was conducted by consensus. In these cases (n = 9), the third measurement was used as the final result.

### Statistical Analyses

Descriptive data are presented as the means ± standard deviation. A categorical comparison was performed using the χ^2^ or Fisher exact test. A receiver operating characteristic (ROC) curve of the SUVmax and GGO ratio was generated to obtain the cutoff value that yielded optimal sensitivity and specificity for the prediction of recurrence. A multivariate logistic regression analysis was used to identify independent risk factors for the patients with recurrence. The predictive value of the established model was assessed by calculating the area under the ROC curve (AUC). According to Hanley and McNeil,^18^ an AUC value of 0.51 to 0.60 indicates the absence of discrimination, 0.61 to 0.70 indicates poor discrimination, 0.71 to 0.80 indicates acceptable discrimination, 0.81 to 0.90 indicates good discrimination, and 0.91 to 1.0 indicates perfect discrimination. The RFS curves were assessed using the Kaplan–Meier method, and the differences between the variables were determined using the log-rank test. A multivariate Cox proportional hazards model was used to determine independent variables for predicting RFS. Two-sided *P* values <0.05 were considered statistically significant. All statistical analyses were performed using SPSS software (version 17.0; SPSS, Chicago, IL) and MedCalc (MedCalc Software, Ostend, Belgium).

## RESULTS

### Patient Characteristics

The characteristics of the 145 enrolled patients are shown in Table 1. The majority of the patients received lobectomies (n = 132; 91%). The mean diameter of the tumors was 1.98 cm (range, 0.5–3 cm). The majority of the histological subtypes were adenocarcinoma (n = 131; 90.3%), followed by squamous cell carcinoma (n = 8; 5.5%) and others (n = 6; 4.1%). LVI was identified in 9 patients. During clinical follow-up, 21 (14.5%) patients developed recurrence at the following sites: lung (n = 11), regional lymph node (n = 6), pleura (n = 5), bone (n = 3), and liver (n = 1).

**Table 1:**
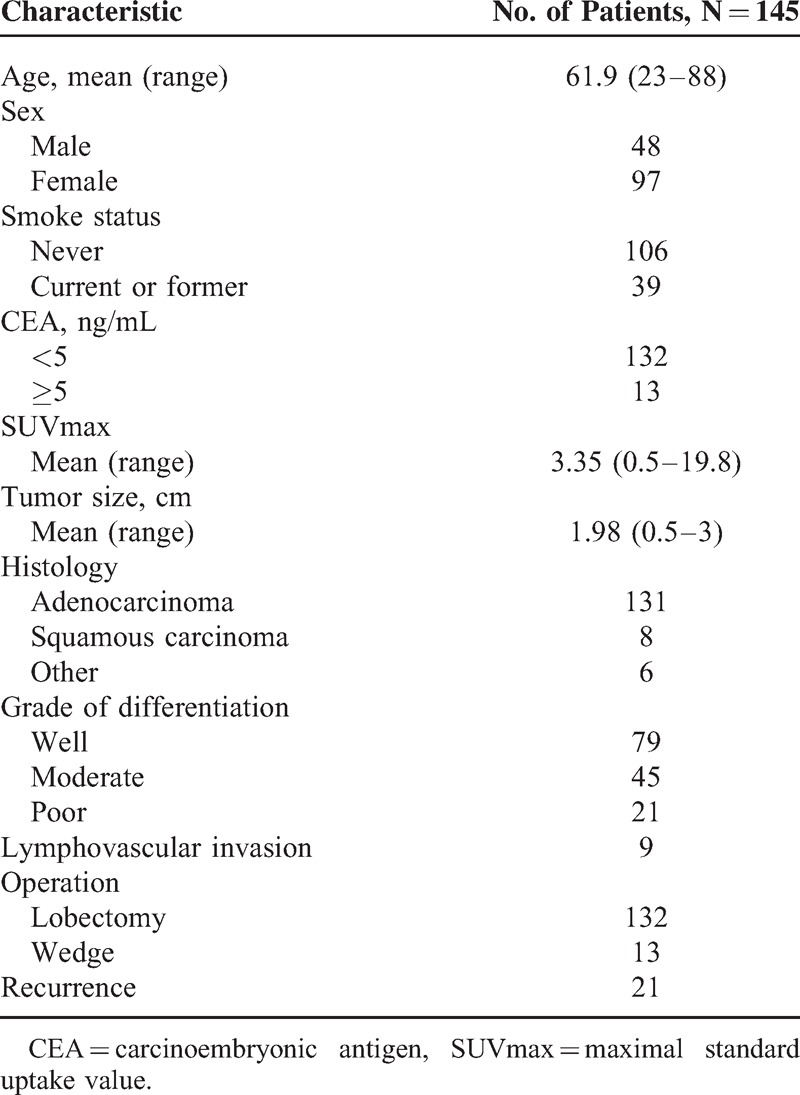
Patient Characteristics

### Association Between Clinical Factors and Recurrence

The ROC curve revealed that the cutoff point of SUVmax was 2.5, and the calculated AUC was 0.725 (95% confidence interval [CI], 0.64–0.79) (acceptable discrimination). We dichotomized the tumors according to this threshold (SUVmax = 2.5), and the sensitivity and specificity for predicting recurrence were 90.5 and 56.5%, respectively. Among the clinical factors, SUVmax and the grade of histological differentiation exhibited a significant correlation with recurrence (Table 2). Recurrence was more frequently identified in patients with a high SUV (≥2.5) compared with in those with a low SUV (<2.5) (20.6% vs 2.7%; *P* < 0.001). Patients with poorly differentiated tumors were more likely to experience recurrence compared with patients with well or moderately differentiated tumors (25% vs 10%; *P* = 0.02). With respect to the LVI, there was no significant difference.

**Table 2:**
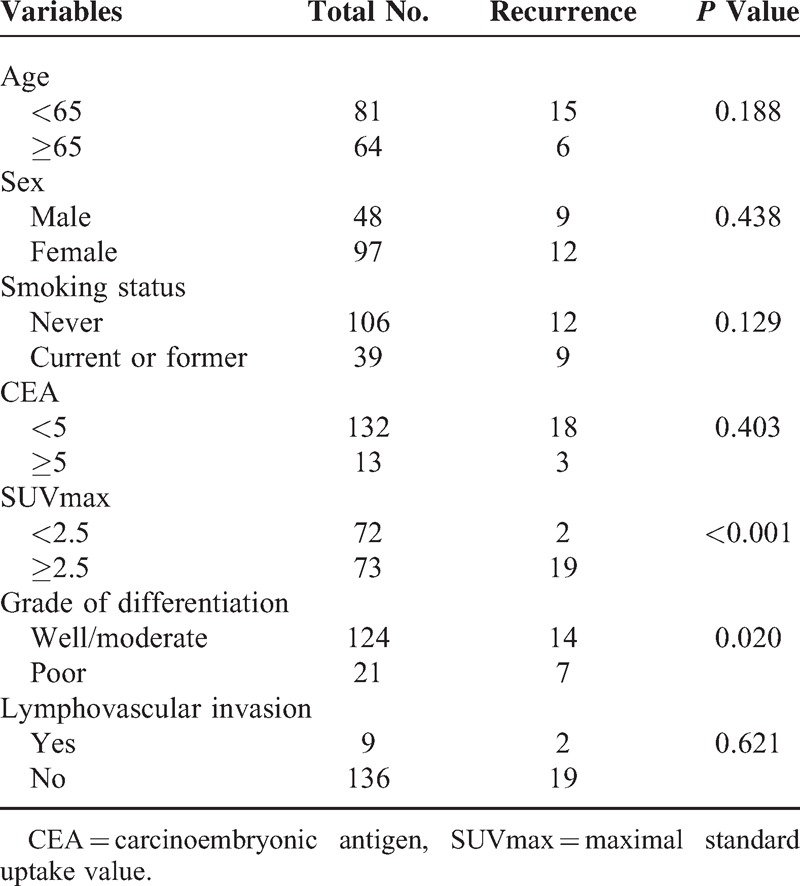
Association Between Clinical Features and Recurrence

### Association Between CT Features and Recurrence

**Figure 1:**
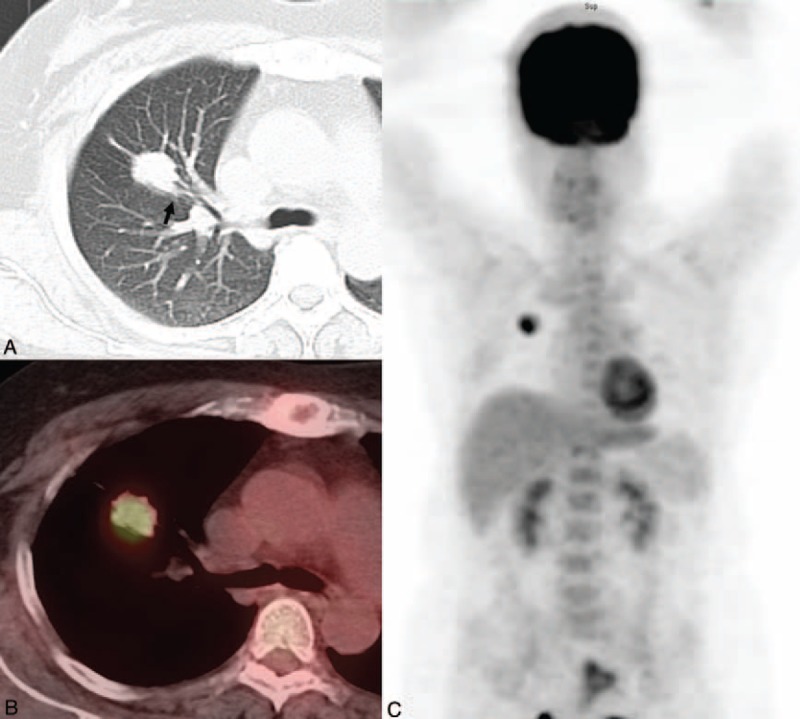
Representative images of a 50-year-old female with a pathological stage IA adenocarcinoma. (A) Lung window of CT scan showing a 2.6-cm lobulated solid tumor with a thickening bronchovascular bundle (arrow) in the right upper lobe. ^18^F-FDG PET/CT in the axial plane (B) and whole-body maximum-intensity-projection (C) images demonstrating high ^18^F-FDG avidity (SUVmax, 5.6) in the primary lesion. The patient encountered tumor recurrence during a 16-month follow-up. CT = computed tomography, FDG = fluorodeoxyglucose, PET = positron emission tomography, SUVmax = maximal standard uptake value.

The ROC analysis revealed that the AUC for the GGO ratio was 0.726 (95% CI, 0.65–0.79) (acceptable discrimination). The optimal cutoff value for the GGO ratio for predicting recurrence was ≤17% (sensitivity, 81.0%; specificity, 58.9%). A comparison between the recurrence and no recurrence groups identified significant differences in the tumor size, the GGO ratio, and the presence of bronchovascular bundle thickening. The patients with recurrence tended to present a larger tumor size (≥2 cm) and lower tumor GGO ratio (≤17%) (*P* = 0.035 and 0.002, respectively). Tumors associated with the presence of bronchovascular bundle thickening were more frequently observed in the recurrent group compared with in the nonrecurrent group (*P* = 0.002) (Figure 1). However, no significant difference was identified between the 2 groups regarding tumor location or other morphologic features (Table 3).

**Table 3:**
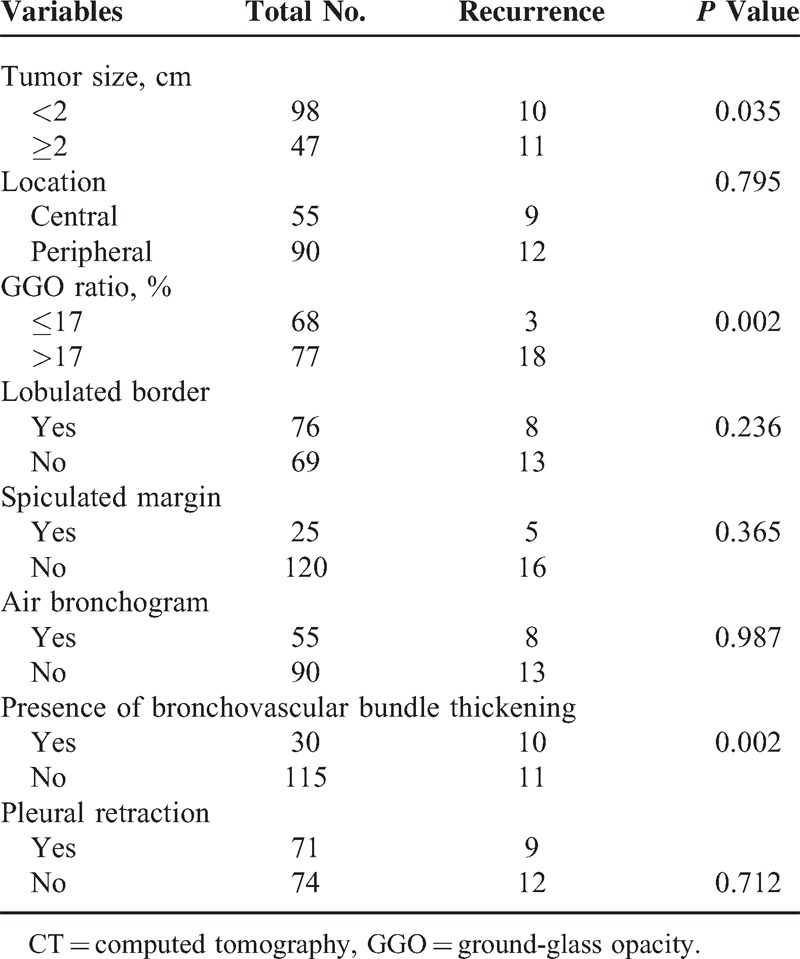
Association Between CT Characteristics and Recurrence

### Multivariate Analysis of Various Factors for the Prediction of Recurrence

**Figure 2:**
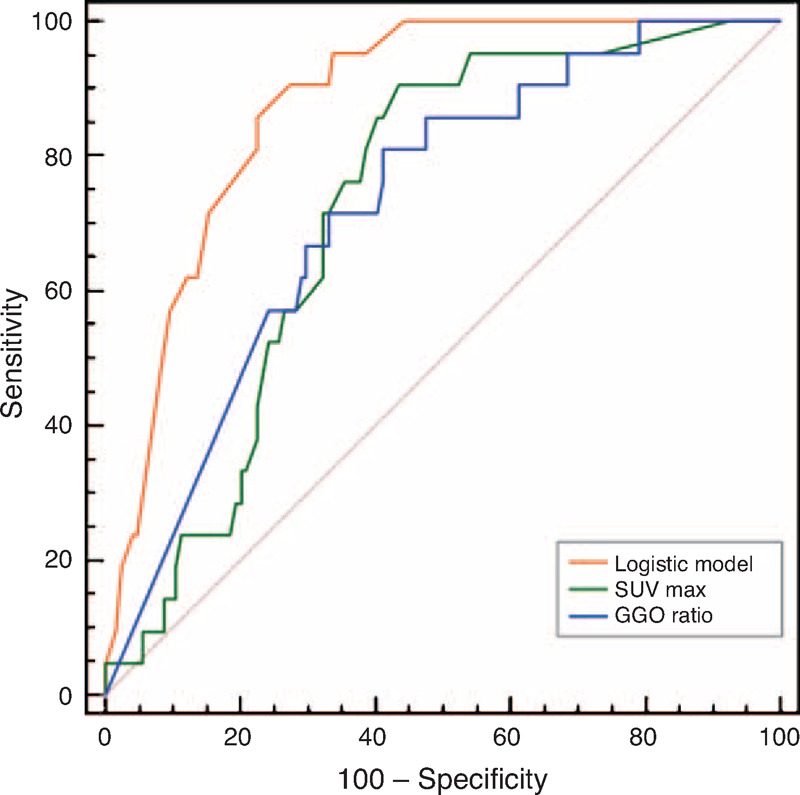
The AUC of the logistic model including SUVmax, the GGO ratio, and the presence of bronchovascular bundle thickening was significantly higher (AUC = 0.877) than the AUC of the SUVmax alone (AUC = 0.725) or GGO ratio alone (AUC = 0.726) (*P* < 0.001). AUC = area under the ROC curve, GGO = ground-glass opacity, SUVmax = maximal standard uptake value.

SUVmax, the grade of histological differentiation, tumor size, the GGO ratio, and the presence of bronchovascular bundle thickening were included in the multivariate logistic regression analysis. The multivariate analysis revealed that a higher SUVmax, a lower GGO ratio, and the presence of bronchovascular bundle thickening were significant independent predictive factors of recurrence (Table 4). An ROC curve analysis was also performed to quantify the performance of this logistic regression model in the prediction of recurrence, and the AUC increased to 0.877 (95% CI: 0.81, 0.93), which suggests that these predictors had good discrimination. The performance of the predictive model using SUVmax, the GGO ratio, and morphologic CT features was significantly higher than using only SUVmax or the GGO ratio alone (*P* < 0.001) (Figure 2).

**Table 4:**
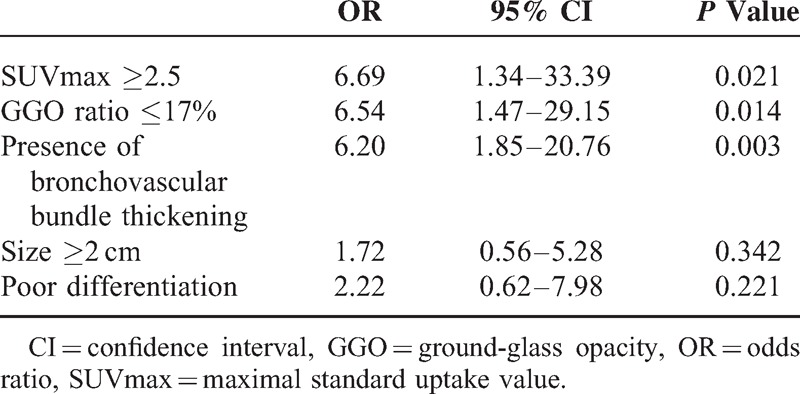
Multivariate Analysis of Various Factors for the Prediction of Recurrence

### Relationship Between Predictive Risk Factors and Recurrence-Free Survival

**Figure 3:**
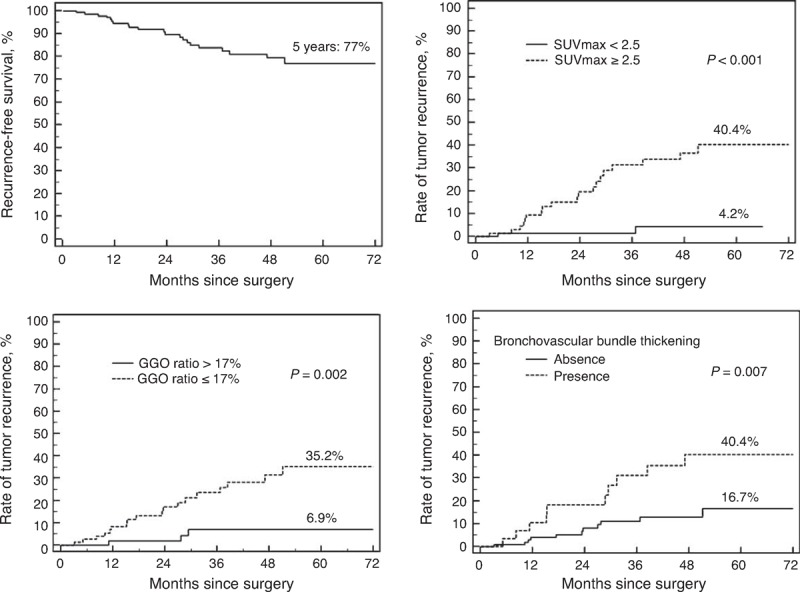
(A) Five-year recurrence-free survival probability of the 145 patients in the study cohort. The cumulative rate of 5-year tumor recurrence according to SUVmax (B), the GGO ratio (C), and the presence of bronchovascular bundle thickening (D). GGO = ground-glass opacity, SUVmax = maximal standard uptake value.

The 5-year RFS rate was 77% in this study cohort, and the median RFS time was 29.3 months (range 3.2–79.4 months). The 5-year RFS was analyzed according to the predictive model (SUVmax, the GGO ratio, and the presence of bronchovascular bundle thickening). The cumulative rate of tumor recurrence was also significantly higher for patients with tumors who presented a high SUVmax (≥2.5), a low GGO ratio (≤17%), and the presence of bronchovascular bundle thickening (log-rank test: *P* < 0.001, =0.002 and 0.007, respectively) (Figure 3). The multivariate Cox proportional analyses also revealed that a high SUVmax, a low GGO ratio, and the presence of bronchovascular bundle thickening were independent prognostic factors for RFS; the hazard ratios were 5.69, 3.83, and 2.69, respectively (Table 5).

**Table 5:**
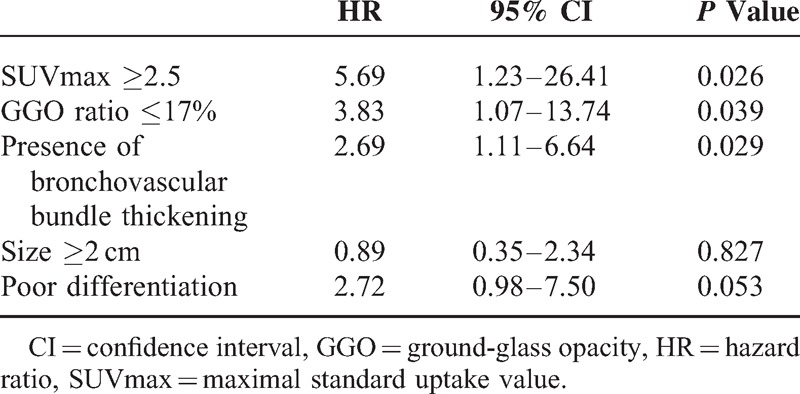
Multivariate Analysis for Recurrence-Free Survival Using Cox Proportional Hazard Model

## DISCUSSION

Previous studies have reported that several clinicopathological factors, such as smoking history, CEA level, surgical type, tumor size, histological differentiation of the tumor, and LVI, were identified as poor prognostic factors and correlated with recurrence in patients with stage I NSCLC.^9,19–21^ Recently, LVI and histological differentiation have become well-recognized prognostic factors in stage I NSCLC, and postoperative adjuvant chemotherapy may improve the survival outcome for this high-risk group.^5,9^ In the current study, we not only evaluated the clinical factors of patients with stage IA NSCLC but also investigated the radiological factors, including FDG uptake and CT imaging features. However, we observed that patients with stage IA NSCLC developed recurrence despite the negative finding of LVI and other clinical factors, including the grade of histological differentiation, which also exhibited no significant difference in the multivariate analysis. Interestingly, based on the results of this study, FDG uptake and CT imaging features are significant risk factors for the recurrence of stage IA NSCLC.

Prior series studies have reported that the preoperative SUVmax has prognostic value in early stage NSCLC and that a higher SUVmax is frequently correlated with recurrence.^7,13,14,22^ Our results are also compatible with these findings, and the cutoff value of SUVmax in our study was identified as 2.5. However, in a review of the literature, variable cutoff values of SUVmax were identified and ranged from 2.9 to 5.5.^7,13,14,22^ The potential reasons for the different values observed between our study and other studies are as follows: first, most previous studies selected patients based on clinical stage. However, the clinical stage of a tumor cannot fully predict its pathological stage, and a more advanced stage correlates with a higher SUV.^23^ Second, the histological types of tumors evaluated in previous studies included more squamous cell carcinomas, which have been shown to have higher FDG uptake compared with adenocarcinoma.^14^ It also must be emphasized that SUVmax is a semiquantitative index that varies between different institutes or PET scanners depending on several parameters, such as fasting duration, level of plasma glucose, time to imaging, reconstruction algorithms, and ROI. Despite the limitations in establishing a standard cutoff value, we demonstrated a trend for a higher SUVmax in cases with recurrence. Furthermore, the main population of our study had adenocarcinoma (90.3%) with pathological stage IA, and the SUVmax cutoff value of 2.5 is close to that reported by Uehara et al and Kishimoto et al.^22,24^ Therefore, we suggest that this value might be an appropriate threshold of early and small adenocarcinoma.

In the evaluation of pulmonary nodules on thin-sliced CT images, previous studies have demonstrated that lower tumor GGO ratios and the presence of bronchovascular bundle thickening are unfavorable prognostic factors and that most tumors with these imaging features usually correlate with pathological invasiveness, such as LVI, a well-established prognostic factor.^16,22,25–27^ However, although the prognostic factors demonstrated in our study were similar to previous studies, most of our patients with recurrence rarely exhibited evidence of LVI in pathological evaluation. In fact, the assessment of LVI may be affected by different staining methods. It has been reported that immunohistochemical staining, such as podoplanin, D2-40, or CD34, is more accurate for the detection of LVI compared with hematoxylin and eosin staining.^28^ Moreover, LVI also might be missed if the vascular lumen has been obliterated by tumor cells. Therefore, based on our results, CT morphologic features still have a role in the prediction of prognosis despite the fact that no invasive finding was identified in the pathological assessment. Furthermore, we suggest that tumors with CT imaging features indicating a lower GGO ratio and the presence of bronchovascular bundle thickening may be stratified as a high-risk group for recurrence.

Although few published studies have evaluated the presence of thickened bronchovascular bundles as a prognostic factor of tumor recurrence, we demonstrated that a morphologic lesion analysis that included this feature enhanced our performance in the prediction of tumor recurrence compared with an analysis that included SUVmax or the GGO ratio alone. When SUVmax or the GGO ratio was applied alone, the AUC was 0.725 and 0.726, respectively. Considering the higher AUC of 0.877 in the multivariate analysis, we believe that including SUVmax, the GGO ratio, and the presence of thickened bronchovascular bundles can increase the predictive ability of the model, especially in comparison with the use of a single parameter alone, to identify patients at high risk of tumor recurrence. Furthermore, this model could be easily applied in clinical practice.

There were some limitations to our study. First, this study was a retrospective design conducted in a single institution and included a relatively small number of patients. A larger, multi-institutional prospective study is needed for further validation of the current results. Second, because our cohort mainly consisted of adenocarcinoma (90.3%), the results of our study may not represent typical stage IA NSCLC. Thus, research to further evaluate the prognostic factors of nonadenocarcinoma groups is warranted. Third, our criteria are based on subjective assessments of CT morphologic features. A CT attenuation-based approach or assessment by means of computerized schemes to improve measurement and interpretation should be made in future studies. Finally, although simplicity is a great advantage of SUVmax measurement, it may not reflect metabolic activity of the entire neoplastic lesion because of the heterogeneity of NSCLC. Recently, several published studies have reported that volume-based PET parameters such as metabolic tumor volume (MTV) and total lesion glycolysis (TLG) were better than SUVmax for predicting prognosis in early stage NSCLC.^29–33^ Therefore, further prospective studies including these parameters will be designed.

In conclusion, our results highlight the predictive value of radiological factors for recurrence in pathological IA NSCLC, especially in the cases of tumors without LVI in pathological evaluation, and we suggest that a higher SUVmax, a lower GGO ratio, and the presence of bronchovascular bundle thickening on CT imaging are correlated with recurrence. The use of this predictive model might be helpful in the selection of patients for postoperative adjuvant chemotherapy or for inclusion in further prospective clinical trials.
